# Fusing similarity rankings in ligand-based virtual screening

**DOI:** 10.5936/csbj.201302002

**Published:** 2013-02-24

**Authors:** Peter Willett

**Affiliations:** a Information School, University of Sheffield, 211 Portobello Street, Sheffield S1 4DP, United Kingdom

**Keywords:** Combination methods, Ranking methods, Similarity measures, Similarity searching, Virtual screening

## Abstract

Data fusion is the name given to a range of methods for combining multiple sources of evidence. This mini-review summarizes the use of one such class of methods for combining the rankings obtained when similarity searching is used for ligand-based virtual screening. Two main approaches are described: similarity fusion involves combining rankings from single searches based on multiple similarity measures; and group fusion involves combining rankings from multiple searches based on a single similarity measure. The review then focuses on the rules that are available for combining similarity rankings, and on the evidence that exists for the superiority of fusion-based methods over conventional similarity searching.

## Introduction

Virtual screening involves ranking a database of previously untested molecules in order of decreasing probability of biological activity, and is an increasingly important component of lead-discovery programmes in the agrochemical and pharmaceutical industries [1–4]. There are two main approaches: *structure-based virtual screening*, which requires knowledge of the 3D structure of the biological target; and *ligand-based virtual screening*, which requires knowledge of at least some ligands that exhibit the desired bioactivity. In this paper, we focus on *similarity searching*, which is arguably the simplest, and probably the most widely, used approach currently available for ligand-based virtual screening [5–9].

In its simplest form, similarity searching assumes the existence of at least one active (or potentially active) molecule, which is normally referred to as the *reference* or *target* structure, and a database of molecules that have not, thus far, been tested in the assay of interest. If one assumes that molecules that are structurally similar are likely to have similar properties, an assumption that is normally referred to as the *similar property principle* [10], then the molecules most similar to the reference structure are those with the greatest probabilities of activity, and hence prime candidates for biological testing.

There are very many different ways in which inter-molecular similarities can be computed, but all measures comprise three basic components: the *representation* that characterizes each molecule; the *weighting scheme* that is used to (de)prioritise different parts of the representation to reflect their relative importance; and the *similarity coefficient* that provides a numeric value for the degree of similarity between two weighted representations. Many different types of representation have been reported in the literature [7, 8, 11] but these are all of three basic types: sets of computed molecular properties (such as molar volume, molecular weight, numbers of heteroatoms, log octanol/water partition coefficient etc) yielding so-called 1D representations; topological (or 2D) representations encoding patterns of atoms and bonds; and representations that encode 3D atom coordinate or shape information. There have been only limited discussions of weighting schemes for similarity searching [12, 13] but many studies of the various types of representation and similarity coefficient that are available [7, 8, 14–17]. Combining the three components hence enables the creation of very large numbers of possible similarity measures, with several detailed comparisons available that seek to establish the most appropriate for chemical similarity searching [8, 18–20]. However, it has become widely recognised that no single measure can be expected to provide the best level of search effectiveness in all circumstances [2, 18, 21–23], with the result that researchers have looked for ways of combining the results obtained from use of multiple similarity searches. This is normally effected using the technique known as *data fusion* [24]; an analogous combination approach, there called *consensus scoring*, is also widely used in structure-based virtual screening [25].

## Data fusion

The term ‘data fusion’ is used to describe a range of methods for combining information that has been obtained in digital form from different sources, with the aim of producing a fused source that is more informative than are individual data sources [26–28]. The techniques are used in many different application areas [29]. When used for similarity searching, a data source is a similarity measure that calculates a similarity score for each of the structures in a database and then ranks the structures in decreasing order of these scores, where the scores (or the ranks, *vide infra*) are assumed to reflect the probabilities of each of the database structures exhibiting the same biological activity as the reference structure. The availability of multiple sources of information means that combining several different similarity rankings to give a single fused ranking is expected to provide a superior level of screening effectiveness than will the ranking obtained from any single similarity measure.

The basic procedure that has been developed for similarity searching is shown in algorithmic form below.

FOR *x*:= 1 to *n* DO

FOR *y*:= 1 to *N* DO

Calculate the similarity, *SIM*_*x*_(*d*_*y*_), for the *y*-th database-structure using the *x*-th similarity scoring function

FOR *y*:= 1 to *N* DO

Use a fusion rule, *F*, to combine the set of *n* scores {*SIM*_*x*_(*d*_*y*_)} for the *y*-th database-structure to give its fused score, *FSIM*_*y*_ Sort the database into decreasing order of the fused scores, *FSIM*_*y*_

**Figure 1:**
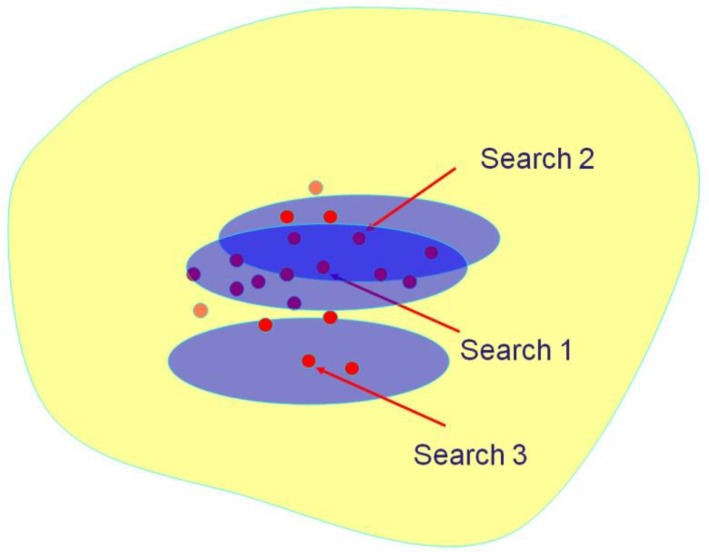
Individual search outputs for three similarity searches (the purple ovals) of a chemical database (the yellow volume), with highly similar active molecules denoted by the red circles.

**Figure 2:**
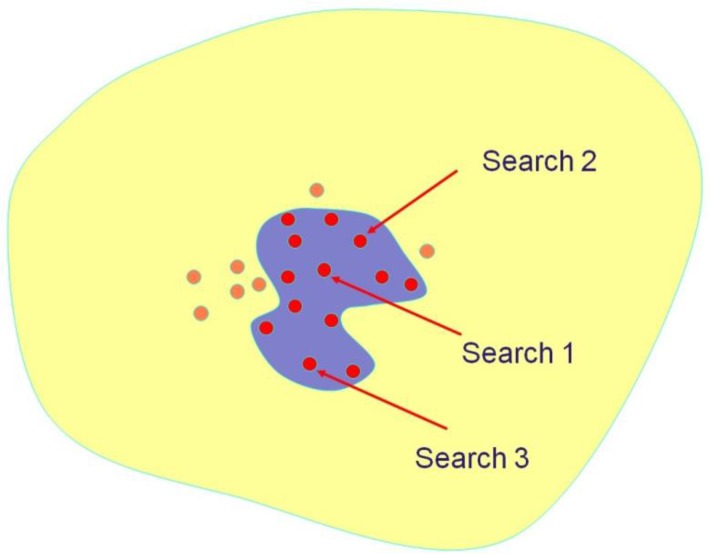
Combined search output resulting from the application of a fusion rule to the three individual search outputs in Figure 1.

In this algorithm, there are *n* different ways for calculating the similarity *SIM*_*x*_(*d*_*y*_) for each of the *N* structures in the database that is being searched (1 ≤ *x* ≤ *n*, and 1 ≤ *y* ≤ *N*). The fusion rule, *F*, is a procedure that combines the set of *n* different similarity scores for each database structure, *y*, to a yield the final fused score, *FSIM*_*y*_. The *N* fused scores, one for each database structure, are then sorted into decreasing order to provide the final output of the similarity search. The procedure is shown diagrammatically in Figures 1 and 2. The yellow shading denotes the database that is to be searched and the purple ovals in Figure 1 denote the sets of top-ranked molecules retrieved in three individual similarity searches, e.g., those occurring in the top-1% of the rankings. Some of these retrieved molecules are active, as denoted by the red circles. Figure 2 shows the application of a fusion rule to the three individual search outputs, with the resulting combined output, e.g., the top-1% of the fused ranking, containing a greater concentration of actives than do the outputs in Figure 1 from the three individual searches.

The fusion procedure, as described digrammatically in the figures or algorithmically in the pseudo-code is completely general in nature and can be implemented in several different ways.

First, one must specify the nature of the *n* different searches that are carried out [30]: in *similarity fusion*, *n* different similarity measures are used to search the database with a single reference structure; and in *group fusion*, *n* different reference structures are used to search the database with a single similarity measure. The similarity fusion approach was the first to be discussed in the late Nineties. Sheridan *et al*. at Merck described the fusion of pairs of rankings generated using different types of fingerprint [31, 32] while Ginn *et al*. at Sheffield described the fusion of 2D, 3D and spectral rankings generated using different types of similarity coefficient [33, 34]. Both groups found that data fusion gave search results that were generally at least as effective as the best individual similarity searches, and that multiple sources of information could lessen the rather substantial variations in effectiveness that are often encountered in individual searches using conventional approaches to similarity-based screening. The group-fusion approach was first studied in detail by Willett *et al*. at Sheffield, comparing the results obtained with those from similarity fusion and from conventional similarity searching [30, 35, 36]. They found that group fusion was notably superior to the other two approaches, especially when searching for structurally heterogeneous sets of active molecules, and group fusion has become widely used as standard technique for ligand-based virtual screening [7].

Second, one must specify what is fused once the searches have been carried out. The algorithm above assumes that it is the actual similarities, i.e., the set of *n* scores {*SIM*_*x*_(*d*_*y*_)} for the *y*-th database-structure, that are combined to give the fused score that forms the basis for the final ranking that is presented to the user. Alternatively, the fusion rule can be applied to the ranks of the *N* database structures when all of the similarity scores are ranked in decreasing order [30, 34, 37]. Ranks are derived from similarities and hence provide less information; however, they are useful in similarity fusion when, as is often the case, the *n* different similarity measures give similarity scores that follow non-identical frequency distributions and that could hence introduce some degree of bias into the results. For example, if similarities are calculated using the cosine coefficient and the Tanimoto coefficient, which are two of the best-known and most widely used similarity coefficients [38], then the cosine scores will always be greater than the Tanimoto scores (except at the extremal values of zero and unity, when the two will be identical).

Thus far, we have referred to the combination of different rankings, so as to produce a single output ranking, without specifying how the combination is achieved in practice. This is the function of a *fusion rule*, and many such rules have been reported in the literature as described in the following section.

## Fusion rules

Using the notation in the algorithm above, the basic input to a fusion rule comprises *n* (*n* ≥ 2) sets of *N* similarities or ranks and the output is a ranking of the *N* structures comprising the database that is being searched. The many fusion rules that have been discussed in the literature are of two basic types: an *unsupervised* rule operates directly on the similarity or rank information, whereas a *supervised* rule requires an initial training procedure using available structure-activity data. In this mini-review we focus on the former class of rules since they have been more widely used to date; however, we shall exemplify the latter class by describing work on belief theory carried out by a group at Abbott [39].

Fusion is normally implemented by applying simple arithmetic operations on the lists of similarity scores (or ranks) resulting from the *n* searches, and these arithmetic fusion rules are reviewed in some detail by Chen *et al*. [40]. The two most common examples of this class are the so-called MAX and SUM rules. Using the terminology in the algorithm shown previously, the fused score *FSIM*_*y*_ for the MAX rule has the form

Max{SIM_1_(d_y_), SIM_2_(d_y_)..SIM_x_(d_y_)..SIM_n_(d_y_)},

i.e., it assigns the *y*-th database-structure, *d*_*y*_, a score that is the largest of the *n* similarities to the reference structure that have been calculated; while the fused score for the SUM rule has the form

$$\sum_{x=1}^{n}SIM_{x}(d_{y})$$

and hence assigns *d*_*y*_ a score that is the sum (or, equivalently, the arithmetic mean) of the *n* individual similarities. An early comparison of arithmetic rules for similarity fusion by Ginn *et al*. suggested that the SUM rule was generally the most effective [34]. However, Hert *et al*. found that the MAX rule was notably more effective for group fusion when similarity scores were to be fused [35, 41]. This finding was confirmed in a very detailed comparative study by Nasr *et al*. that used over 40 public datasets [42] and the approach has now been widely adopted (see, e.g., [7, 43–45]).

Although defined above in terms of similarity scores, *SIM*_*x*_(*d*_*y*_), such arithmetic rules are equally applicable to the rank data, *RANK*_*x*_(*d*_*y*_), obtained when the similarity scores are sorted into descending order. Chen *et al*. describe a further rule, the reciprocal rank fusion (RRF) rule, that is applicable only to rank data and that derives from the fact that virtual screening often involves applying a cut-off on the similarity scores (such as the top-1%) so that only a small fraction of the database is considered further in a project [40]. Let *p* (*p* ≤ *n*) be the number of times that an individual database structure *d*_*y*_, occurs above the chosen cut-off. Then the RRF rule involves summing the reciprocal ranks for those *p* occurrences to give a fused score

$$\sum_{x=1}^{p}\frac{1}{RANK_{x}(d_{y})}.$$

Chen *et al*. found that RRF out-performed all of the other rules that they considered in their detailed comparative study. They ascribed this to the close relationship they were able to demonstrate between the reciprocal rank of a database structure and its probability of activity as determined from an analysis of sets of bioactive molecules in the MDDR and WOMBAT databases.

Another, more complex fusion rule has been described recently by Cross *et al*. [46]. Fusion here is based on Pareto ranking, where the Pareto rank of each database structure is the number of structures that have a larger similarity score in all of the *n* ranked lists that are to be fused. Ties in this initial Pareto ranking are then resolved by considering the number of molecules with larger similarities in all but one (all but two, all but three etc.) ranked lists, a procedure that Cross *et al*. found to be superior to the SUM rule when used with rank data.

Unsupervised fusion rules, such as those described above, require just the *n* sets of *N* similarity scores (or the resulting ranks) as inputs, whereas the many supervised rules additionally require a quantitative relationship between the structural similarity of two molecules and their corresponding similarities in activity. Several such approaches have been described [45, 47–49] as exemplified by the recent study of Muchmore *et al*. on data fusion using *belief theory* [39]. This involves the calculation of a degree of belief in some outcome given the evidence available from different sources, i.e., belief in the activity of a database structure given its similarities to the reference structure in a set of similarity searches. Muchmore *et al*. analysed a large in-house file of screening data to identify the similarities, using various similarity measures, between pairs of molecules that had comparable activities, and were hence able to derive a relationship between *B*_*x*_, the belief that a pair of molecules are equally active using the *x*-th similarity measure, and *SIM*_*x*_, the similarity score for the *x*-th similarity measure. The rule for combining the individual beliefs for a given database structure in each of the *n* similarity searches is

$$1-\prod_{x=1}^{n}(1-Bx),$$

and Muchmore *et al*. found that this rule yielded rankings that were comparable to those resulting from use of the SUM rule but that were easier to interpret [39]. The approach has subsequently been used for lead-hopping [50] and for combining the results of ligand-based and structure-based virtual screening [51].

The increasing availability of large volumes of linked chemical and biological data means that supervised fusion rules are likely to become more widely used in the future; currently, unsupervised rules provide a simple, widely used approach to the effective combination of multiple search outputs.

## Why does data fusion work?

The basic assumption in data fusion is that the availability of multiple sources of information (i.e., similarity rankings in the present context) will yield better results than when just a single source is available. The review by Willett [24] summarized a range of studies demonstrating that this does indeed seem to be the case for ligand-based screening: fusion-based screening is often comparable with, or even superior to, the best of the screening methods that are being combined, especially when group fusion is used; and fusion results in a level of screening effectiveness that is far more consistent from search to search than is the case when just a single similarity method is available. Studies in Sheffield have investigated the reasons for the success of data fusion, using both empirical and theoretical approaches [52–55].

Whittle *et al*. developed and tested an analytical model of fusion-based similarity searching [52–54]. The study focused on the use of the SUM and MAX rules in similarity fusion to combine pairs of rankings derived from searches with different similarity coefficients, but Whittle *et al*. demonstrated that their methods could be extended to similarity fusion with different types of fingerprint or to group fusion. Assume that searches are carried out using two similarity coefficients, such as the cosine coefficient and the Tversky coefficient, and that one then plots the corresponding frequency distributions for the similarities between the reference structure and the database structures. Consider the numbers of similarities that are of magnitude at least *x* using the cosine coefficient and at least *y* using the Tversky coefficient: an effective fusion rule will then be one that preferentially populates this portion of the joint frequency distribution with active molecules (or depopulates it with inactive molecules) when compared with the corresponding distributions for each of the individual coefficients. Whittle *et al*. demonstrate that this is the case in practice for the SM and MAX fusion rules if, and only if, sufficient training data are available, since even the fusion of just two similarity lists requires information about eight distinct frequency distributions. If some cut-off, e.g., the top-1%, is applied to each ranking then the following distributions must be considered: those for the top-ranked actives and for the top-ranked inactives above the cut-off for each similarity coefficient for both the database structures that occur above the cut-off in both lists and for those occurring in just one of the lists. When such data are available then the model predicts that the MAX rule will perform better than the SUM rule for group fusion, that SUM will be better than MAX for similarity fusion, and that the former type of fusion is generally to be preferred. These predictions are fully in accord with the many previous empirical studies [24], hence validating the model and providing a rationale for why data fusion can indeed enhance the effectiveness of similarity searching. However, the model's complexity and the volume of training information that it requires means that it is most unlikely that it could be used, as was originally the hope, to predict the utility of new types of fusion rule.

Drawing on work carried out by Spoerri on the use of data fusion to combine the outputs of text search engines [56], Holliday *et al*. have reported a systematic study of the use of multiple rankings for similarity-based virtual screening [55]. Their experiments used two standard test databases, the MDDR and WOMBAT databases [57], and similarity searches with five different similarity coefficients and five different types of fingerprint, i.e., a total of 25 different similarity measures. A similarity search was carried out for a bioactive reference structure using one of these measures and a note taken of the number of top-ranked database structures that had the same bioactivity as the reference structure (specifically, a database structure was assumed to have been retrieved in a screening search if it occurred in the top-1% of the ranking after the database had been ranked in order of decreasing similarity with the reference structure). This procedure was repeated for each of the other 24 similarity measures, so that it was possible to determine how many database structures were retrieved by just one measure, by just two measures, by just three measures etc. It was found that very many structures were retrieved in the top-1% of a single search but that the numbers of retrieved structures fell away very rapidly as one considered the top-1% of two searches, of three searches, of four searches etc. This behaviour was observed consistently across all the types of bioactivity that were searched for, suggesting that this is an entirely general phenomenon. Indeed, Holliday *et al*. were able to demonstrate and to rationalise the existence of a power law relationship [58, 59] between the numbers of structures retrieved and the numbers of searches. Since there are decreasingly few structures common to increasing numbers of rankings, then data fusion will be effective when many of these common structures have the same bioactivity as the reference structure. Holliday *et al*. showed that only a small proportion of the many structures retrieved by a single search were active, but that this proportion increased rapidly as one considered the structures retrieved by two searches, the structures retrieved by three searches etc. The probability of activity of a database structure hence increases in line with its frequency of retrieval in multiple similarity searches, thus providing a simple, but direct, empirical justification for using combination methods to enhance the effectiveness of virtual screening.

## Summary and outlook

Similarity searching is one of the most widely used methods for ligand-based virtual screening. A range of different types of similarity measure are available for this purpose, and data fusion provides a simple way of combining the results from multiple similarity searches to increase the effectiveness of screening above that normally obtainable from the use of a single similarity measure. Two approaches to fusion have been described in the literature: similarity fusion involves matching a single reference structure against a database using multiple similarity measures; while group fusion involves matching multiple reference structures against a database using a single similarity measure. If multiple actives are available then the latter procedure is normally to be preferred.

The fusion rules that have attracted most attention thus far are unsupervised, in the sense that they do not require any training data relating similarity scores to probabilities of activity; however the increasing availability of such structure-activity data means that supervised rules provide an obvious focus for future research in data fusion. Other areas where developments may be expected include the combination of different types of virtual screening method, the comparison of supervised fusion with existing screening approaches based on machine learning (which also requires the availability of extensive training data), and further attempts to provide a theoretical underpinning for the use of fusion methods.

## References

[1] Alvarez J, Shoichet B, editors (2005) *Virtual Screening in Drug Discovery*. Boca Raton: CRC Press

[2] McGaughey GB, Sheridan RP, Bayly CI, Culberson JC, Kreatsoulas C et al (2007) Comparison of topological, shape, and docking methods in virtual screening. J Chem Inf Model 47: 1504–19 17591764 10.1021/ci700052x

[3] Rippenhausen P, Nisius B, Peltason L, Bajorath J (2010) Quo vadis, virtual screening? A comprehensive survey of prospective applications. J Med Chem 53: 8461–7 20929257 10.1021/jm101020z

[4] Schneider G (2010) Virtual screening: an endless staircase? Nature Rev Drug Discov 9: 273–6 20357802 10.1038/nrd3139

[5] Eckert H, Bajorath J (2007) Molecular similarity analysis in virtual screening: foundations, limitation and novel approaches. Drug Discov Today 12: 225–33 17331887 10.1016/j.drudis.2007.01.011

[6] Ripphausen P, Nisius B, Bajorath J (2011) State-of-the-art in ligand-based virtual screening. Drug Discov Today 16: 372–6 21349346 10.1016/j.drudis.2011.02.011

[7] Stumpfe D, Bajorath J (2011) Similarity searching. WIRES Comp Mol Sci 1: 260–82

[8] Willett P (2009) Similarity methods in chemoinformatics. Ann Rev Inf Sci Technol 43: 3–71

[9] Willett P (2011) Similarity-based data mining in files of two-dimensional chemical structures using fingerprint-based measures of molecular resemblance. WIRES Data Mining Knowledge Disc 1: 241–51

[10] Johnson MA, Maggiora GM, editors (1990) *Concepts and Applications of Molecular Similarity*. New York: John Wiley

[11] Todeschini R, Consonni V (2002) *Handbook of Molecular Descriptors*. Weinheim: Wiley-VCH

[12] Arif SM, Holliday JD, Willett P (2009) Analysis and use of fragment occurrence data in similarity-based virtual screening. J Comput Aid Mol Design 23: 655–68 10.1007/s10822-009-9285-0 19536456

[13] Arif SM, Holliday JD, Willett P (2010) Inverse frequency weighting of fragments for similarity-based virtual screening. J Chem Inf Model 50: 1340–9 20672867 10.1021/ci1001235

[14] Gower JC, Legendre P (1986) Metric and Euclidean properties of dissimilarity coefficients. J Classif 5: 5–48

[15] Hubálek Z (1982) Coefficients of association and similarity, based on binary (presence-absence) data: an evaluation. Biol Rev Cambridge Phil Soc 57: 669–89

[16] Holliday JD, Hu C-Y, Willett P (2002) Grouping of coefficients for the calculation of inter-molecular similarity and dissimilarity using 2D fragment bit-strings. Combin Chem High Through Screen 5: 155–66 10.2174/1386207024607338 11966424

[17] Chen X, Reynolds CH (2002) Performance of similarity measures in 2D fragment-based similarity searching: comparison of structural descriptors and similarity coefficients. J Chem Inf Comput Sci 42: 1407–14 12444738 10.1021/ci025531g

[18] Bender A, Jenkins JL, Scheiber J, Sukuru SCK, Glick M, Davies JW (2009) How similar are similarity searching methods? A principal components analysis of molecular descriptor space. J Chem Inf Model 49: 108–19 19123924 10.1021/ci800249s

[19] Duan J, Dixon SL, Lowrie JF, Sherman W (2010) Analysis and comparison of 2D fingerprints: Insights into database screening performance using eight fingerprint methods. J Mol Graph Model 29: 157–70 20579912 10.1016/j.jmgm.2010.05.008

[20] Sastry M, Lowrie JF, Dixon SL, Sherman W (2010) Large-scale systematic analysis of 2D fingerprint methods and parameters to improve virtual screening enrichments. J Chem Inf Model 50: 771–48 20450209 10.1021/ci100062n

[21] Sheridan RP, Kearsley SK (2002) Why do we need so many chemical similarity search methods? Drug Discov Today 7: 903–11 12546933 10.1016/s1359-6446(02)02411-x

[22] Sheridan RP (2007) Chemical similarity searches: when is complexity justified? Expert Opin Drug Discov 2: 423–30 23484752 10.1517/17460441.2.4.423

[23] Bender A (2010) How similar are those molecules after all? Use two descriptors and you will have three different answers. Expert Opin Drug Discov 5: 1141–51 22822717 10.1517/17460441.2010.517832

[24] Willett P (2006) Data fusion in ligand-based virtual screening. QSAR Combin Sci 25: 1143–52

[25] Feher M (2006) Consensus scoring for protein-ligand interactions. Drug Discov Today 11: 421–8 16635804 10.1016/j.drudis.2006.03.009

[26] Hall DL, McMullen SAH (2004) *Mathematical Techniques in Multisensor Data Fusion*. Norwood MA: Artech House

[27] Liggins ME, Hall DL, Llinas J, editors (2008) *Handbook of Multisensor Data Fusion: Theory and Practice*. Boca Raton FL: CRC Press

[28] Mitchell HB (2007) *Multi-Sensor Data Fusion: An Introduction*. Berlin: Springer

[29] Dasarathy BV (2010) A representative bibliography of surveys in the information fusion domain. Inf Fusion 11: 299–300

[30] Whittle M, Gillet VJ, Willett P, Alex A, Loesel J (2004) Enhancing the effectiveness of virtual screening by fusing nearest neighbor lists: A comparison of similarity coefficients. J Chem Inf Comput Sci 44: 1840–8 15446844 10.1021/ci049867x

[31] Kearsley SK, Sallamack S, Fluder EM, Andose JD, Mosley RT et al (1996) Chemical similarity using physicochemical property descriptors. J Chem Inf Comput Sci 36: 118–27

[32] Sheridan RP, Miller MD, Underwood DJ, Kearsley SK (1996) Chemical similarity using geometric atom pair descriptors. J Chem Inf Comput Sci 36: 128–36

[33] Ginn CMR, Turner DB, Willett P, Ferguson AM, Heritage TW (1997) Similarity searching in files of three-dimensional chemical structures: evaluation of the EVA descriptor and combination of rankings using data fusion. J Chem Inf Comput Sci 37: 23–37

[34] Ginn CMR, Willett P, Bradshaw J (2000) Combination of molecular similarity measures using data fusion. Perspect Drug Discov Design 20: 1–16

[35] Hert J, Willett P, Wilton DJ, Acklin P, Azzaoui K et al (2004) Comparison of fingerprint-based methods for virtual screening using multiple bioactive reference structures. J Chem Inf Comput Sci 44: 1177–85 15154787 10.1021/ci034231b

[36] Hert J, Willett P, Wilton DJ, Acklin P, Azzaoui K et al (2006) New methods for ligand-based virtual screening: use of data-fusion and machine-learning techniques to enhance the effectiveness of similarity searching. J Chem Inf Model 46: 462–70 16562973 10.1021/ci050348j

[37] Yang J-M, Chen Y-F, Shen T-W, Kristal BS, Hsu DF (2005) Consensus scoring criteria for improving enrichment in virtual screening. J Chem Inf Model 45: 1134–46 16045308 10.1021/ci050034w

[38] Willett P, Barnard JM, Downs GM (1998) Chemical similarity searching. J Chem Inf Comput Sci 38: 983–96

[39] Muchmore SW, Debe DA, Metz JT, Brown SP, Martin YC et al (2008) Application of belief theory to similarity data fusion for use in analog searching and lead hopping. J Chem Inf Model 48: 941–8 18416545 10.1021/ci7004498

[40] Chen B, Mueller C, Willett P (2010) Combination rules for group fusion in similarity-based virtual screening. Mol Informatics 29: 533–41 10.1002/minf.201000050 27463331

[41] Hert J, Willett P, Wilton DJ, Acklin P, Azzaoui K et al (2004) Comparison of topological descriptors for similarity-based virtual screening using multiple bioactive reference structures. Org Biomol Chem 2: 3256–66 15534703 10.1039/B409865J

[42] Nasr RJ, Swamidass SJ, Baldi PF (2009) Large scale study of multiple-molecule queries. J Cheminf 1: 7 at http://www.jcheminf.com/content/1/1/7 10.1186/1758-2946-1-7 PMC3225883 20298525

[43] Williams C (2006) Reverse fingerprinting, similarity searching by group fusion and fingerprint bit importance. Mol Diversity 10: 311–32 10.1007/s11030-006-9039-z 17031535

[44] Hristozov DP, Oprea TI, Gasteiger J (2007) Virtual screening applications: a study of ligand-based methods and different structure representations in four different scenarios. J Comput Aid Mol Design 21: 617–40 10.1007/s10822-007-9145-8 18008169

[45] Tiikkainen P, Markt P, Wolber G, Kirchmair J, Distinto S et al (2009) Critical comparison of virtual screening methods against the MUV data set. J Chem Inf Model 49: 2168–78 19799417 10.1021/ci900249b

[46] Cross S, Baroni M, Carosati E, Benedetti P, Clementi S (2010) FLAP: GRID molecular interaction fields in virtual screening. Validation using the DUD data set. J Chem Inf Model 50: 1442–50 20690627 10.1021/ci100221g

[47] Raymond JW, Jalaie M, Bradley PP (2004) Conditional probability: a new fusion method for merging disparate virtual screening results. J Chem Inf Comput Sci 44: 601–9 15032541 10.1021/ci034234o

[48] Baber JC, Shirley WA, Gao Y, Feher M (2006) The use of consensus scoring in ligand-based virtual screening. J Chem Inf Model 46: 277–88 16426063 10.1021/ci050296y

[49] Tiikkainen P, Poso A, Kallioniemi O (2009) Comparison of structure fingerprint and molecular interaction field based methods in explaining biological similarity of small molecules in cell-based screens. J Comput Aid Mol Design 23: 227–39 10.1007/s10822-008-9253-0 19050828

[50] Martin YC, Muchmore S (2009) Beyond QSAR: lead hopping to different structures. QSAR Combin Sci 28: 797–801

[51] Swann SL, Brown SP, Muchmore SW, Patel H, Mert P et al (2011) A unified, probabilistic framework for structure- and ligand-based virtual screening. J Med Chem 54: 1223–32 21309579 10.1021/jm1013677

[52] Whittle M, Gillet VJ, Willett P, Loesel J (2006) Analysis of data fusion methods in virtual screening: theoretical model. J Chem Inf Model 46: 2193–205 17125164 10.1021/ci049615w

[53] Whittle M, Gillet VJ, Willett P, Loesel J (2006) Analysis of data fusion methods in virtual screening: similarity and group fusion. J Chem Inf Model 46: 2206–19 17125165 10.1021/ci0496144

[54] Whittle M, Gillet VJ, Willett P (2010) A simulation study of the use of similarity fusion for ligand-based virtual screening In: Lodhi H, Yamanishi Y editors. *Chemoinformatics and Advanced Machine Learning Perspectives: Complex Computational Methods and Collaborative Techniques*. Hershey PA: IGI Global

[55] Holliday JD, Kanoulas E, Malin N, Willett P (2011) Multiple search methods for similarity-based virtual screening: analysis of search overlap and precision. J Cheminf 3: 29 at http://www.jcheminf.com/content/3/1/29 10.1186/1758-2946-3-29 PMC3195112 21824430

[56] Spoerri A (2008) Authority and ranking effects in data fusion. J Amer Soc Inf Sci Tech 59: 450–60

[57] Gardiner EJ, Gillet VJ, Haranczyk M, Hert J, Holliday JD et al (2009) Turbo similarity searching: Effect of fingerprint and dataset on virtual-screening performance. Stat Anal Data Mining 2: 103–14

[58] Newman MEJ (2005) Power laws, Pareto distributions and Zipf's law. Contemp Phys 46: 323–51

[59] Benz RW, Swamidass SJ, Baldi P (2008) Discovery of power-laws in chemical space. J Chem Inf Model 48: 1138–51 18522387 10.1021/ci700353m

